# Top-Down Analysis of Temporal Hierarchy in Biochemical Reaction Networks

**DOI:** 10.1371/journal.pcbi.1000177

**Published:** 2008-09-12

**Authors:** Neema Jamshidi, Bernhard Ø. Palsson

**Affiliations:** Department of Bioengineering, University of California San Diego, La Jolla, California, United States of America; University of Tokyo, Japan

## Abstract

The study of dynamic functions of large-scale biological networks has intensified in recent years. A critical component in developing an understanding of such dynamics involves the study of their hierarchical organization. We investigate the temporal hierarchy in biochemical reaction networks focusing on: (1) the elucidation of the existence of “pools” (i.e., aggregate variables) formed from component concentrations and (2) the determination of their composition and interactions over different time scales. To date the identification of such pools without prior knowledge of their composition has been a challenge. A new approach is developed for the algorithmic identification of pool formation using correlations between elements of the modal matrix that correspond to a pair of concentrations and how such correlations form over the hierarchy of time scales. The analysis elucidates a temporal hierarchy of events that range from chemical equilibration events to the formation of physiologically meaningful pools, culminating in a network-scale (dynamic) structure–(physiological) function relationship. This method is validated on a model of human red blood cell metabolism and further applied to kinetic models of yeast glycolysis and human folate metabolism, enabling the simplification of these models. The understanding of temporal hierarchy and the formation of dynamic aggregates on different time scales is foundational to the study of network dynamics and has relevance in multiple areas ranging from bacterial strain design and metabolic engineering to the understanding of disease processes in humans.

## Author Summary

Cellular metabolism describes the complex web of biochemical transformations that are necessary to build the structural components, to convert nutrients into “usable energy” by the cell, and to degrade or excrete the by-products. A critical aspect toward understanding metabolism is the set of dynamic interactions between metabolites, some of which occur very quickly while others occur more slowly. To develop a “systems” understanding of how networks operate dynamically we need to identify the different processes that occur on different time scales. When one moves from very fast time scales to slower ones, certain components in the network move in concert and pool together. We develop a method to elucidate the time scale hierarchy of a network and to simplify its structure by identifying these pools. This is applied to dynamic models of metabolism for the human red blood cell, human folate metabolism, and yeast glycolysis. It was possible to simplify the structure of these networks into biologically meaningful groups of variables. Because dynamics play important roles in normal and abnormal function in biology, it is expected that this work will contribute to an area of great relevance for human disease and engineering applications.

## Introduction

The network of interactions that occur between biological components on a range of various spatial and temporal scales confer hierarchical functionality in living cells. In order to determine how molecular events organize themselves into coherent physiological functions, in silico approaches are needed to analyze how physiological functions emerge from the evolved temporal structure of networks. Time scale decomposition is a well-established, classical approach to dissecting network dynamics and there is a notable history of analyzing the time scale hierarchy in metabolic networks and matching the events that unfold on each time scale with a physiological function [1]–[6]. This approach enables the identification of the independent, characteristic time scales for a dynamic system. In particular it has been possible to decompose a cell-scale kinetic model of the human red blood cell in time to show how its key metabolic demands are met through a dynamic structure-function relationship. The underlying principle is one of aggregation of concentration variables into ‘pools’ of concentrations that move in tandem on slower time scales [5],[7].

The dynamics of biological networks characteristically span large time scales (8 to 10 orders of magnitude), which contributes to the challenge of analyzing and interpreting related models. However, there is structure in this dynamic hierarchy of events, particularly in biochemical networks in which the fastest motions generally correspond to the chemical equilibria between metabolites, and the slower motions reflect more physiologically relevant transformations. Appreciation of this observation can result in elucidating structure from the network and simplifying the interactions. The reduction in dynamic dimensionality is based on such pooling and the analysis of pooling is focused in the underlying time scale hierarchy and its determinants. Understanding the time scale hierarchy and pooling structure of these networks is critical to understanding network behavior and simplifying it down to the core interactions.

Top-down studies of dynamic characteristics of networks begin with fully developed kinetic models that are formal representations of large amounts of data about the chemistry and kinetics component interactions. Network properties can be studied by numerical simulations (that are condition-specific) or by analysis (that often yield general model properties) of the model equations. Since comprehensive numerical simulation studies become intractable for larger networks and the identification of general model properties are needed for the judicious simplification of models, there is a need for analysis based methods in order to characterize properties of dynamic networks. In this study we present an in silico analysis method to determine pooling of variables in complex dynamic models of biochemical reaction networks. This method is used to study metabolic network models and allows us to identify and analyze pool formation resulting from the underlying stoichiometric, thermodynamic, and kinetic properties.

## Results/Discussion

### Method Development

#### Dynamic description of networks

Linearizing the mass conservation equations for a chemical reacting system around the steady state yields the linear form of the dynamic mass balances,
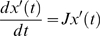
(1)where *J* is the **n**×**n** Jacobian matrix, and *x*′ ( = *x*−*x*_ss_) is the deviation vector of the concentration variables from the steady state (*x*_ss_). *J* describes the dynamical characteristics of the network near the steady state. The properties of *J* can be analyzed using matrix decomposition methods, and it is important for these methods to capture the interactions between components across all of the time scales of the network.

#### Temporal decomposition

We can apply a similarity transformation (see Materials and Methods) to *J*,
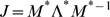
(2)where Λ is a diagonal matrix with the eigenvalues, that are the negative reciprocal time constants [8]. The superscript star indicates the possible presence of imaginary components in the matrices. Complex conjugate pairs can be removed by pre-multiplying by a modified identity matrix with block diagonal ones at the rows and columns in which the matrix has imaginary components. The complex conjugate pairs arise in situations in which the motions of these modes cannot be decomposed in time. The modal matrix, *M* ^−1^, can then be used to define the modes, *m*, such that,
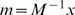
(3)which combined with Equation 1 results in,
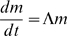
(4)as described previously [5]. The rows in the modal matrix define dynamically independent “aggregates”, or pooling of metabolites into independent dynamic variables. Time scale decomposition can be successfully performed only if the eigenvalues are well separated. When *J* is rank deficient, it implies the presence of dynamically invariant pools reflecting chemically conserved moieties in the network, whose sum total is constant. These vectors are not included in the modal matrix.

**Figure 1:**
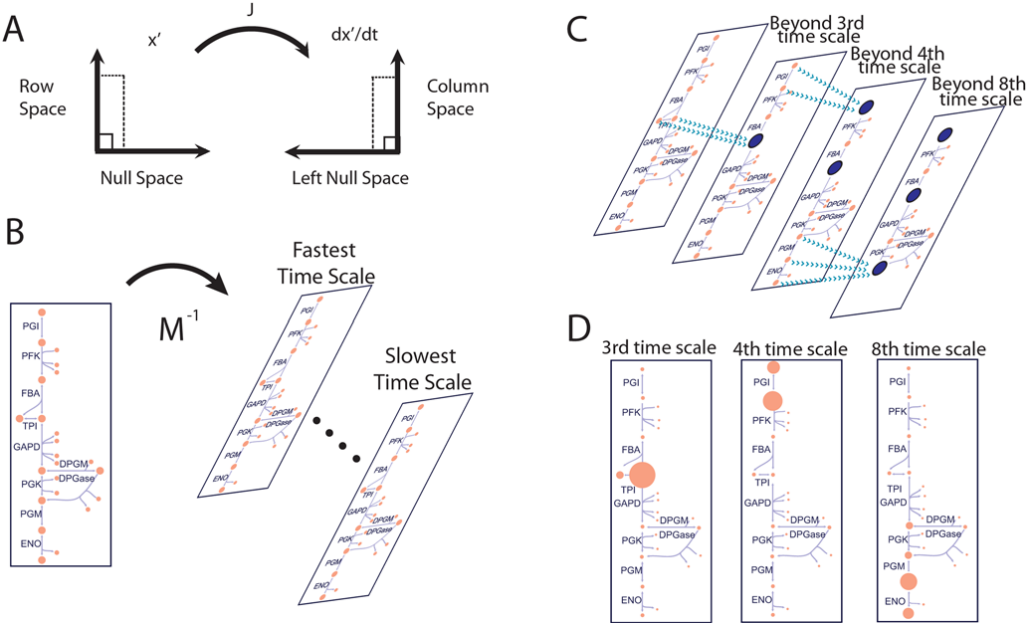
Subspaces of the Jacobian matrix and different approaches for decomposing it into dynamically independent interactions between metabolites. (A) The Jacobian acts as a linear operator mapping the dynamics onto the deviation variable. (B) The Modal Matrix maps network dynamics onto independent time scales. Panels C and D illustrate two approaches to understanding the interactions between metabolites on the different time scales. (C) Beginning from the fastest time scale and moving forward, components that move together on subsequent time scales are lumped into an aggregate pool variable. The pooling pictorially for three different time scales in glycolysis and the Rapoport-Leubering shunt in the red cell. The large blue dots indicate pool formation between two metabolites, signifying that these two metabolites become coupled or correlated on slower times scales. In this case, glyceraldehyde 3-phosphate and dihydroxyacetone phosphate pool together after the first time scale, the hexose phosphates pool together after the fourth time scale, and the triose phosphates pool together after the eighth time scale. (D) Each time scale is analyzed independently and the interactions are defined in terms of the coefficients in the model and their contribution to the cumulative sum of the modal coefficients. Analyzing all of the modes in this manner allows the identification of variables that are dominant across multiple modes and identifying the time scales across which they are most active. Four fundamental subspaces are associated with *J* and its mapping onto its time derivative. The key to temporal decomposition is the time-ordered removal of dynamic motions that lead to the step-by-step increase in the null and left null spaces of *J*.

The modal matrix separates the dynamics of the network into a series of dynamically independent motions [5],[8], moving from the fastest (top) modes to the slowest (bottom) modes as the time scales lengthen (Figure 1B). Here we present an approach to the analysis of the modal matrix that simplifies the elucidation of notable pools without the need for intensive calculations of dynamic phase portraits and auto-correlations functions in order to identify biologically relevant interpretations of these modes as pools of metabolites being created and consumed on different time scales [2]. An illustrative ‘toy’ example of time scale decomposition is described in the supplementary material (Figure S1).

Table 2 summarizes some of the trade-offs between the characterization of dynamics using the Jacobian matrix and carrying out large numbers of dynamic simulations directly. Although carrying out dynamic simulations are not restricted to a particular steady state, they are condition dependent (e.g. initial conditions) and resource intensive. Hence for larger networks, dynamic simulations are not a viable option. In contrast, characterization of the pooling structure of networks via analysis of the Jacobian requires only a single set of calculations to characterize a particular steady state and this approach can be applied to large and small networks alike. Furthermore, different steady states can be characterized as well, by recalculating the Jacobian at the alternate steady states.

#### Defining pools from modes

**Figure 2:**
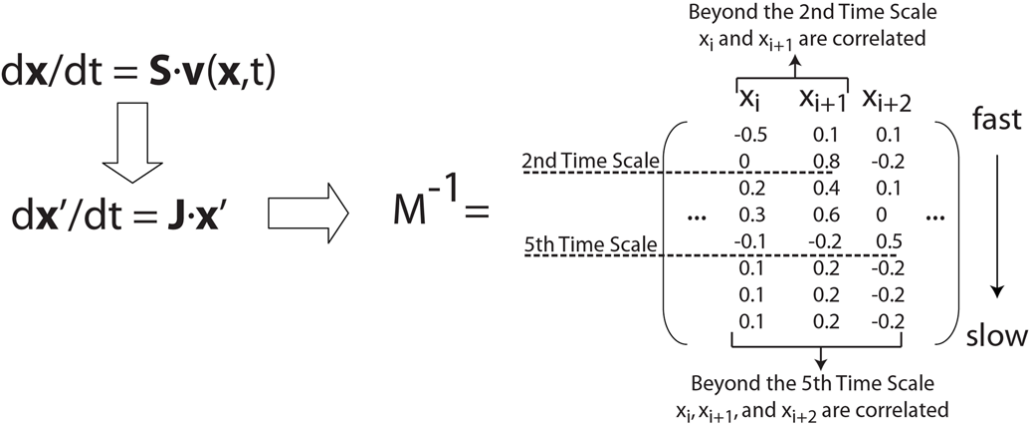
Illustration of the progression correlation between two variables as fast time scales are removed. The set of dynamic equations are linearized about a particular steady state. Applying a similarity transformation to the Jacobian, enables the calculation of the modal matrix (depicted to the right). The rows of the matrix correspond to different time scales. When the ratios between two entries are constant, the two metabolites pool together. After the second time scale, metabolites *x_i_* and *x_i_*_+1_ form an aggregate pool and after the fifth time scale, *x_i_*_+2_ joins the pool. If the variables were compared across all time scales, no significant correlations would be observed. Hence, this simple example highlights the need for a method to analyze correlations between metabolites with consideration of the characteristic time scales of the network.

A column of the modal matrix describes the participation of a concentration in each of the linearly independent modes. When two concentrations (*x_i_* and *x_j_*) become dynamically correlated beyond a particular time scale (say after the *k*th time constant), the entries of the modal matrix in the two corresponding columns are correlated with one another on the subsequent time scales (Figure 2). This characteristic enables the identification of the time scales that two concentrations would pool together.

Employing a geometric interpretation for this determination, one can explicitly identify pool formation by calculating the angle between columns (*M* ^−1^)*_i_* of the modal matrix,
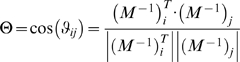
(5)in which 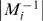 refers to the magnitude of the *i*th column of the modal matrix and *ϑ_ij_* refers to the angle between the *i*th and *j*th columns of the modal matrix. However, if one were to simply calculate the correlations between metabolites across all time scales, in general no pooling would be observed among the metabolites, even though there may be physiologically relevant pooling between metabolites, that characteristically occur on slower time scales. A simple illustration of this is depicted in by the modal matrix in Figure 2, which highlights the need to characterize aggregate pool formation of variables in the context of progressively slower time scales. Hence this approach analyzes progressive pooling across all of the network's independent time scales, in contrast to simulation based methods which are dependent on a priori specification of the time scales of interest for identifying correlations between metabolites.

In order to identify the time scales at which pool formation occurs, we compute the angle between two columns as a function of an index *k* that runs from 1 to *n* time scales. As each row of the modal matrix is removed (*k* increases by one) the angle is recomputed to form a series of angles as a function of *k*; i.e., *ϑ_ij_*(*k*), *k* = 1, 2, …, *n*. If the angle *ϑ_ij_*(*k*) is close to zero, the two columns are correlated at and above that *k* value and the two corresponding concentrations will move in tandem for the subsequent time scales, thus forming an aggregate variable or a pool. The practical issue is to determine when the angle is close enough to zero to make a call on the formation of a pool.

The pools can be described as a sum of matrix products over the time scales of the network:
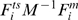
(6)

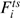
 is a binary diagonal matrix, with *n*−*i* non-zero elements on the diagonal. 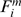 is a binary matrix with off-diagonal elements whenever two columns meet a specific cutoff and can be combined into an aggregate pool. 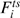 and 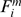 act on the modal matrix, by filtering out modes or combining variables, respectively. For a network with *m* metabolites, in which no aggregate pools form, there will be *m* sets of pooling matrices. Conversely, for the extreme case in which all of the metabolites form a single aggregate pool on a single time scale there will only be a single pooling matrix.

#### Defining dominant interactions for each mode

One can quantitatively ascertain the contribution of each metabolite to each mode by rank ordering the normalized mode and keeping only the largest weights that add up to the specified cutoff percentage. At low cut-off ranges, all metabolites with small contributions to the mode will be zeroed out. The interactions across the modes can be mapped on top of the interactions defined by the stoichiometric matrix in order to compare and contrast the topological connectivity versus the dynamic connectivity at time scales of interest.

### Application of the Method

**Figure 3:**
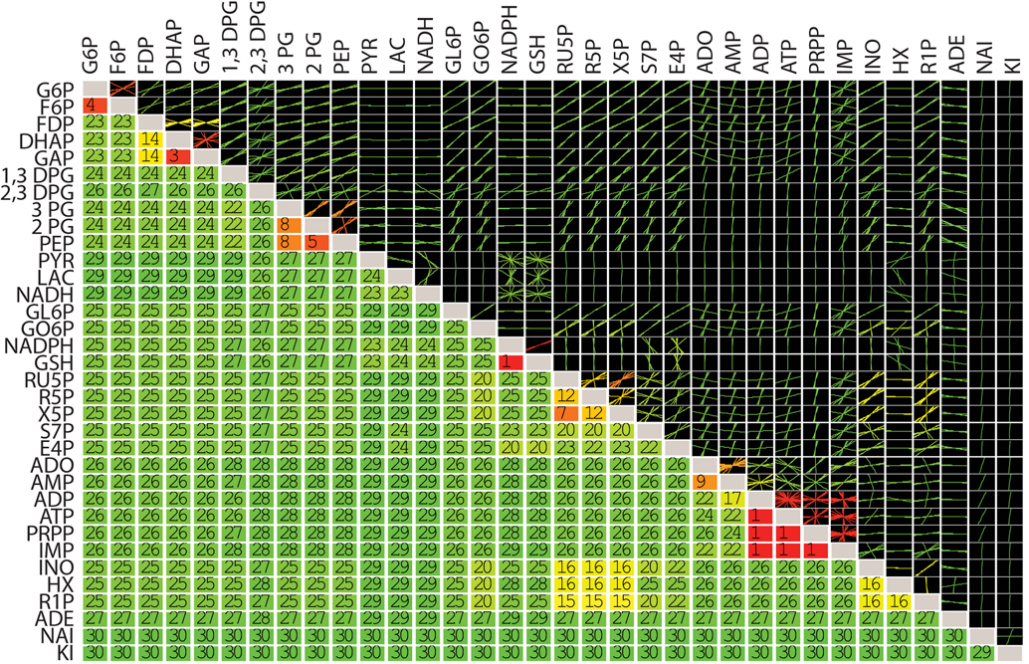
Time scale hierarchy of metabolic pool formation in the human red blood cell. The lower left triangle indicates the modes after which pooling occurs between the corresponding metabolites (one being the fastest time scale). The upper right triangle are plots of the slopes between the two metabolites for the remaining time scales after pool formation (the origin is always included in these approximations of the slopes), color coded according to the time scale at which pooling occurs. Some of the metabolites such as the phosphoglycerates have fairly constant ratios once they join aggregate pools. Others, such as ATP and ADP have varying ratios. These ratios change when interactions with other pathways dominate on subsequent time scales, for example when glycolytic intermediates dominate on one time scale and nucleotide salvage metabolites dominate on another, the respective interactions between ATP and ADP are affected differently. The cutoff value for cos(θ) was 0.9. Abbreviations: G6P, Glucose-6-phosphate; F6P, Fructose-6-phosphate; FDP. Fructose-1,6-bisphosphate; DHAP, Dihydroxyacetone phosphate; GAP, Glyceraldehyde-3-phosphate; DPG13. 1,3-bisphosphoglycerate; DPG23, 2,3-bisphosphoglycerate; PG3, 3-phosphoglycerate; PG2, 2-phosphoglycerate; PEP, Phosphoenolpyruvate; PYR, Pyruvate; LAC. Lactate; NADH, Nicotinamide adenine dinucleotide (reduced); GL6P. 6-Phospho-d-glucono-1,5-lactone; GO6P, 6-Phospho-d-gluconate; NADPH, Nicotinamide adenine dinucleotide phosphate (reduced); GSH, glutathione (reduced); RU5P, Ribulose-5-phosphate; R5P, Ribose-5-phosphate; X5P, Xylulose-5-phosphate; S7P, Sedoheptulose-7-phosphate; E4P, Erythrose-4-phosphate; ADO, Adenosine; AMP, Adenosine monophosphate; ADP, Adenosine diphosphate; ATP, Adenosine triphohsphate; PRPP, 5-Phospho-d-ribose 1-diphosphate; IMP, Inosine monophosphate; INO, Inosine; HX, Hypoxanthine; R1P, Ribose-1-phosphate; ADE, Adenine; NAI, Sodium; and KI, Potassium.

The models studied here exhibit a significant span of time scales (Table 1). A hierarchy pool formation on different time scales was found in all networks based on the calculation of all pair wise *ϑ_ij_*(*k*) in the models (Figures 1C and 2). The results can be presented in a symmetric correlation tiled array, where each entry can be used to represent *k* for a pair of concentrations. Figure 3 shows the result of such an array for the human red cell. Since the array is symmetric we can display both *k* and the modal coefficient ratio in the pool (*x_i_*/*x_j_*) for each pair of concentrations; thus

**Table 1:** Summary of properties of the various kinetic models, particularly the Jacobian matrices of the networks.

System	Dimension	Rank	Pooling	Conservation Pools	Effective Dimensionality	Time Scale Span
**RBC**	34	34	Complete	0	17	1.30E+10
**Folate**	10	9	Complete	1	6	4.88E+04
**Yeast**	20	20	Fragmented	0	13	7.50E+06

Pooling of the tiled modal arrays can be classified as complete (in which all elements pools together eventually) or fragmented. The number of conservation pools is equal to the size of the left null space of the Jacobian. The effective dynamic dimensionality is the number of different time scales at which pooling occurs. The time scale span is the ratio of the largest to smallest eigenvalue for each of the networks.

**Table 2:** A comparison between the trade-offs for analyzing the Jacobian around a particular steady state versus carrying out dynamic simulations.

Jacobian Analysis	Dynamic Simulations
Generalized results	Conditions specific results
Scaleable	Intractable as the number of variables increase
A single set of calculations will characterize a particular steady state	Resource intensive, requires many simulations in order to characterize network pooling
Linear regime near a particular steady state	May move from one steady state to another

The approach presented here for analysis of the Jacobian in order to characterize network dynamics allows generalized, comprehensive elucidation of dynamics around a particular steady state directly and in a scaleable manner. In contrast, although the approach using dynamic simulations is not restricted to a particular steady state, it is resource intensive and for larger networks it is infeasible to characterize all of the different possible initial conditions, due to combinatorial growth of the possible combinations.

**Figure 4:**
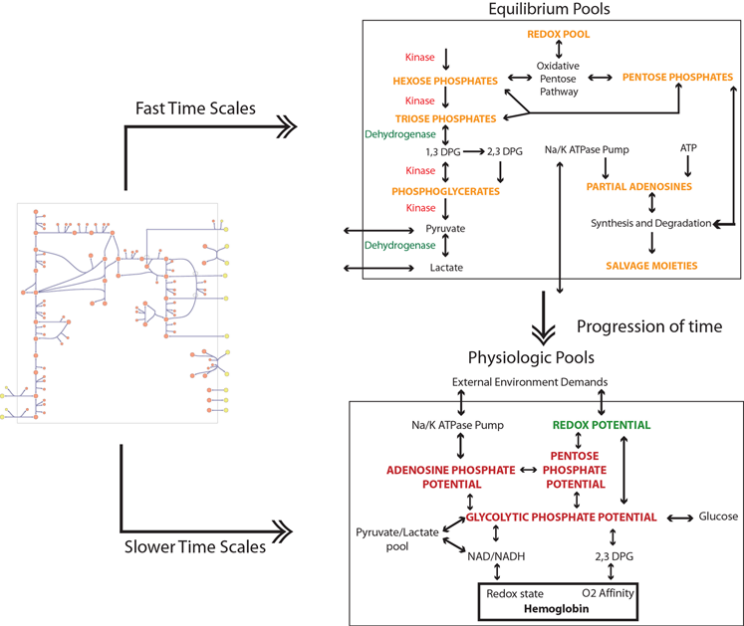
Converting human red cell metabolic network map into lumped pools according to time scale decomposition (adapted from [26]). Pooling on fast time scales define the chemical equilibrium pools and on slower time scales the physiological pools.

- The lower left triangle of the tiled array indicates the time scale *k* beyond which pooling occurs in the network. So for example, G6P and F6P pool together after the fourth mode in the red cell metabolic model. For the highly interconnected metabolic network in the red cell, eventually, all of the metabolites pool together.
- The upper right triangle of the tiled array contains plots show the ratios of the modal coefficients (*x_i_*/*x_j_*) for each of the concentration pairs at all of the time scales above *k* for that pair. For many of the concentration pairs, the ratio remains fairly constant (glycolytic pools, pentose phosphate pools, etc) past a certain time scale showing the relative contribution of the compounds to a pool.
- The pooling structure observed in Figure 3 is consistent with the previous descriptions [2] and enable the simplification of the network into equilibrium pools on fast time scales and physiological ones on slower time scales (Figure 4).

The time scale (*k*) for the formation of pools and the ratio between a pair of concentrations are functions of three factors: network stoichiometry (or topology), thermodynamics, and kinetic properties of the transformations in the network. Viewing the dynamics of the network in terms of the modal matrix and the pair-wise concentration correlations on progressing time scales enables one to consider the questions of (A) the thermodynamic versus kinetic control of concentrations within the whole network and (B) the delineation of kinetic versus topological decoupling in networks.

#### Steady state kinetic versus thermodynamic equilibrium effects

The thermodynamic equilibrium pools of the network can be seen in Figure S2. Comparison of these pools with those in Figure 1 distinguishes between the equilibrium state and the kinetically driven steady state. In many cases such comparisons show a thermodynamic basis for pool formation, such as the hexose phosphates, the pentose phosphates, and the triose phosphates. For metabolite pairs that are thermodynamically close (with regards to the Gibbs free energy of formation), the kinetics presumably have been adjusted to be fast, making such thermodynamically neutral events lead to reduction in effective dynamic dimensionality. Deviations from such behavior are however observed, such as with 1,3 DPG and 2,3 DPG. Although the free energy of formation of these two must are close (as approximated by the group contribution method [9]), 2,3DPG does not pool together with 1,3DPG until the 31^st^ mode (Figure 3). Hence the kinetic and allosteric regulatory “control” dominates. This “control” has physiological significance because 2,3DPG can regulate the binding affinity of hemoglobin for oxygen and is maintained at a higher concentration than the glycolytic intermediates. Other examples in which kinetic “control” dominates include ATP and NADPH, ADP and NADH, and G6P and ADP.

#### Kinetic and topological decoupling

Two striking features of the tiled array are (1) the pooling between the majority of the compounds occurs on the slowest time scales and (2) the slopes for many of these are horizontal or vertical lines, implying dynamically independent behavior. This dynamically independent behavior may result from a lack of connectivity (topological decoupling) or from independent kinetics (e.g., kinetic decoupling resulting from a zero order rate law). Thus, if compounds are detected to be topologically decoupled in the tiling array they are expected to dominate a particular mode.

A kinetic or topologically decoupled compound will undergo the largest changes in concentration and interactions with other compounds during those time scales on which it plays a dominant role the modes. After these time scales have been passed, concentration changes are likely to be less significant and the compounds could be viewed as joining an aggregate pool, but may not be in a fixed ratio as would be dictated through strictly thermodynamic or kinetic coupling.

Networks that are tightly connected in terms of stoichiometry and kinetics will result in complete pooling of all metabolites on the slowest time scales, which is seen in large part in the red cell (Figure 1). There are examples of effectively uncoupled metabolites in this model however. Sodium and potassium for example are uncoupled from all metabolites except for the adenosine phosphates, which results from topological decoupling, since the only metabolites these ions interact with in the model are ATP and ADP via the Na/K ATPase. In contrast, pyruvate and lactate are decoupled from the rest of glycolysis, even though they are topologically connected to some of them, thus the decoupling is driven by kinetic effects.

The tiled arrays can be used to define the ‘effective dynamic dimensionality’ of the models by counting the number of different time scales during which two or more variables form an aggregate pool. For the networks considered, the effective dynamic dimensionality reduced the dimension by one-third to one-half (see Table 1).

**Figure 5:**
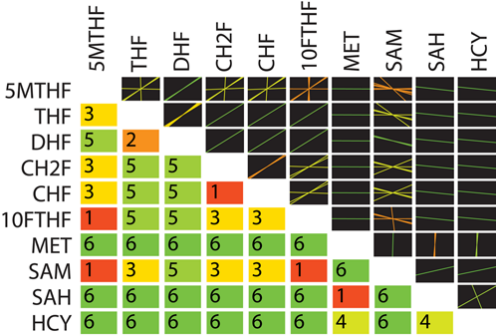
Tiled array of the hierarchical pool formation for human folate metabolism (same layout and color coding as in Figure 3). Abbreviations: 5MTHF, 5-methyltetrahydrofolate; THF, tetrahydrofolate; DHF, dihydrofolate; CH2F, 5,10-methylenetrahydrofolate; CHF, 5,10-methenyltetrahydrofolate; 10FTHF, 10-formyltetrahydrofolate; MET, methionine; SAM, S-adenosylmethionine; SAH, S-adenosylhomocysteine; and HCY, homocysteine.

**Figure 6:**
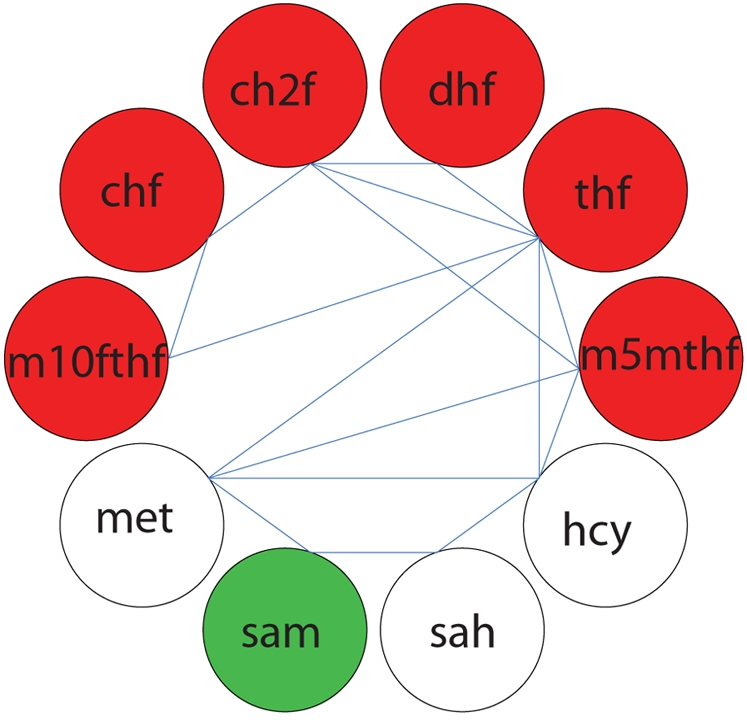
An example of a graphical overlay of topological and kinetic data for the dominant interacting metabolites for a particular mode. The slowest mode in the folate network (∼30 minute time scale) is shown. The green and red shaded elements reflect the dynamic interactions between the metabolites on the 30 minute time scale (the colors reflect positive and negative entries, respectively). The blue lines indicate topological connectivity (i.e. from the stoichiometric matrix).

**Figure 7:**
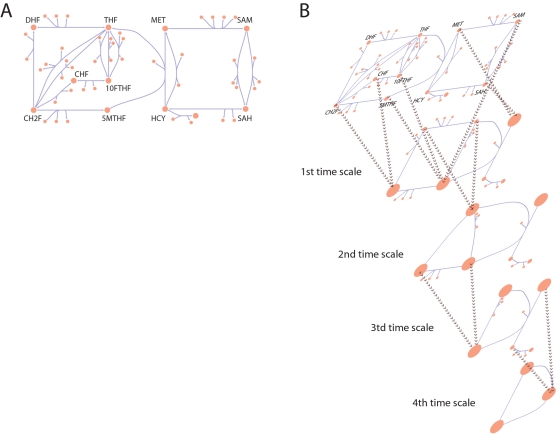
Human folate metabolism and hierarchical simplification into aggregate pools. (A) A map of the folate network described by [23]. (B) An illustration of progressive pool formation across the first 4 time scales for the folate network based on the results from Figure 5. Beyond the first time scale pools form between CHF and CH2F; and 5MTHF, 10FTHF, SAM; and MET and SAH. DHF and THF form a pool beyond the second time scale. Beyond the third time scale CH2F/CHF join the 5MTHF/10FTHF/SAM pool. Beyond the fourth time scale HCY joins the MET/SAH pool. Ultimately, on time scales on the order of a minute and slower, interactions between the pools of folate carriers and methionine metabolites interact.

The tiled pooling array for folate metabolism was computed (Figure 5). There were 7 independent time scales in the modal matrix and one conserved folate moiety pool from the left null space. From the array it is observed that the folate carrier branch (5MTHF, THF, DHF, CH2F, CHF, 10FTHF) of the network and the methionine metabolism branch (MET, SAM, SAH, HCY) of the network act fairly independently dynamically. S-adenosylmethionine (SAM) is the primary metabolite which joins the interactions between the two branches. In order to identify if these interactions are topologically driven, kinetically driven, or combinations of both, one can plot the modes in which these interactions are most significant (see Material and Methods). Figure 6 depicts the primary interactions on the slowest mode in the network. It can be seen that SAM is not directly topologically coupled to the folate branch, so the interactions between the two branches, mediated by SAM is driven by kinetic effects. Additionally, note that although they pool together, SAM moves in opposition to the folate carriers. The folate network is depicted in Figure 7A and the progression of pooling over time is illustrated in Figure 7B. On time scales slower than the minute time scales (the sixth mode corresponds to ∼6 minutes) the network boils down to interactions between the folate pool and the methionine pool. So on physiologically relevant time scales, the various possible interactions depicted in Figure 7A simplify to shifting between two carrier pools.

**Figure 8:**
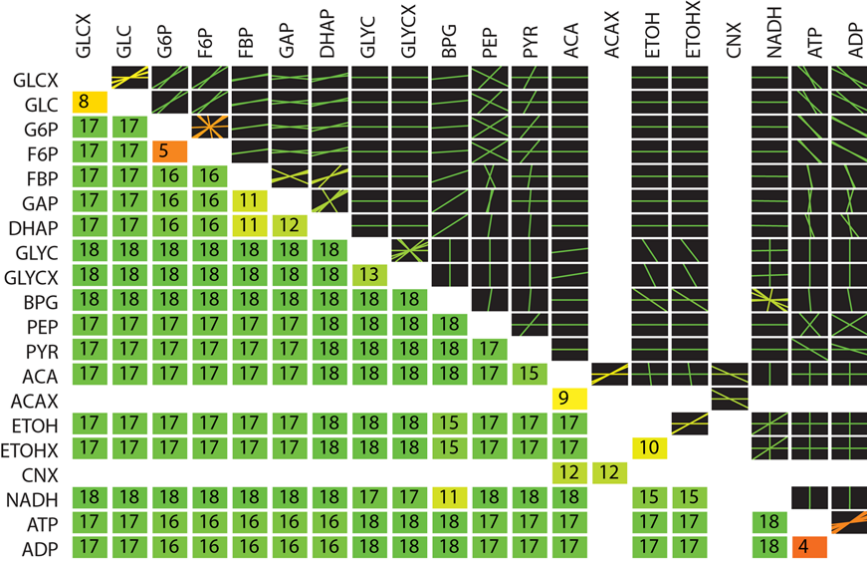
Tiled array of the hierarchical pool formation for the yeast glycolytic pathway (same layout and color coding as in Figure 3). Glycolytic intermediates and adenosine phosphates pool together on fast time scales. Fragmented pooling is also observed (i.e. there were 0 entries in the slowest mode, indicating that on the slowest time scale, all of the components in the network do not move together in a lumped pool). GLC, intracellular glucose; GLCX, extracellular glucose; G6P, glucose 6-phosphate; F6P, fructose 6-phosphate; FBP, fructose 1,6-bisphosphate; GAP, glyceraldehyde 3-phosphate; DHAP, dihydroxyacetone phosphate; GLYC, intracellular glycerol; GLYCX, extracellular glycerol; BPG, 1,3-bisphosphoglycerate; PEP, phosphoenol pyruvate; PYR, pyruvate; ACA, intracellular acetaldehyde; ACAx, extracellular acetaldehyde; EtOH, intracellular ethanol; EtOHx, extracellular ethanol; NADH, nicotinamide adenine dinucleotide (reduced form); ATP. adenosine triphosphate; ADP, adenosine diphosphate.

**Figure 9:**
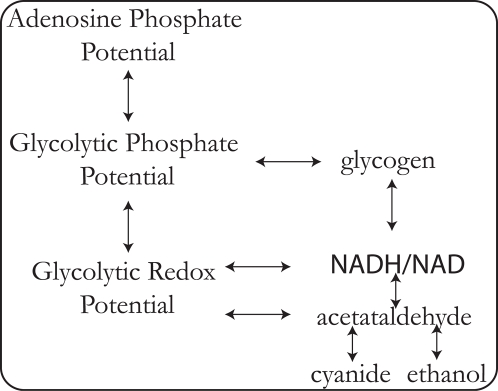
Simplified model of the yeast glycolytic pathway dictated by the fragmented pooling of the network. The glycolytic and redox potentials are similar to those in the red cell. The adenosine phosphate potential is only composed of ATP and ADP. The NADH/NAD ratio determines the redox interactions with glycolysis, glycogen, and conversion between acetaldehyde and ethanol.

The tiled pooling array for the yeast glycolytic pathway was computed (Figure 8). The pooling structure of the glycolytic pathway is very similar to pool formation in the red cell, with glycolytic intermediates aggregating together on the faster time scales. The adenosine phosphates also pool together very quickly. A feature of the tiled array not observed with the other models considered is fragmentation of the pooling structure. This implies that unlike the other two models considered, all of the metabolites do not eventually move together in fixed ratios on the slowest time scales. These effects can be driven by topological properties or kinetic properties of the network as well. Comparing the topological versus kinetic interactions in a graphical format (not shown) illustrates that the lack of interactions by acetate (ACA) and cyanide (CNX) with the other components in the network are due to topological constraints. The fragmented pooling reflects the fact that interactions between ACA and CNX and the rest of the network can only occur during particular time scales. These constraints however dictate much of the overall behavior of the network. The fragmented pooling observed in Figure 8 result in a simplified view of the network built around the transporters, as seen in Figure 9.

Taken together, in this study we: (1) developed top-down approaches for the computationally driven delineation of pools, (2) showed how to distinguish between topological, kinetic and thermodynamic basis for pool formation, and (3) applied the methods to analyze the dynamic structure of metabolic network models in yeast and humans. The application of these methods enabled the simplification of the networks based on the dynamic pooling of metabolites on progressive time scales and the identification of the key metabolic interactions on the slower time scales.

There were some observations in the results worth pointing to suggest further areas worthy of investigation. The pooling ratios between metabolites are not always constant and metabolites that pool early on are more likely to have changing ratios on subsequent time scales. Furthermore, metabolites that are connected to multiple pathways are likely to have change ratios even after they begin pooling. This is observed for ATP and ADP in the human red blood cell (Figure 3). These metabolites interact with glycolysis, the pentose phosphates, and the nucleotide salvage pathway and these interactions vary as the time scales are dominated by the different pathways. Conversely, the ATP/ADP ratio is fairly fixed and uniform in the yeast glycolytic model. This appears to be a result of the fact that there is only one major pathway that interacts with ATP and ADP. Further investigations into when and why pooling ratios change may lead to a richer appreciation of the integrated dynamic structure of metabolic networks. Additionally it was observed that the pooling structure may changes about different steady states. For example, in the human red cell changing the magnitude of the energy load will shift the time scale of the ATPase and hence alter the pooling of the network (not shown). This is also an area in which further investigation will yield an appreciation of altered dynamics corresponding to different steady state conditions.

A growing number of large-scale kinetic models of biochemical reaction networks are becoming available (e.g. http://www.cellml.org/, http://www.siliconcell.net/). There are also growing compilations of information regarding enzyme kinetics (e.g., http://www.brenda-enzymes.info/, http://sabio.villa-bosch.de/SABIORK/), which portend the development and availability of more kinetic models. Since dynamics simulations are not a viable approach for the comprehensive characterization of pool formation for larger models, there is a need for analysis based approaches to identify these general characteristics in metabolic networks.

The pooling approaches developed here were based on identifying the dynamically independent times scales and their corresponding modes. The principle pooling approach considered here was based on a particular calculation given by the matrix product,
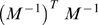
(7)In order to identify pools of metabolites, it was necessary to sequentially eliminate rows and recalculate the product, however it is worth noting that the matrix product in Equation 7 reflects the ‘dynamic connectivity’ of the network. These collective analyses of the modal matrix allowed the subsequent identification of pools, effective dimensionality reduction, differentiation between kinetic and topological properties, and characterizing the component condition numbers and strength of interactions between components in the network.

There has been an increased interest in the analysis of the intrinsic characteristics of the growing number of available large-scale kinetic biological network models [10]–[14]. The systematic, algorithmic approach described herein demonstrated a general approach to pool identification, thus demonstrating how top-down analyses can be used to identify the hierarchical interactions between components over the span of time scales and assist in the simplification, analysis, and interpretation of network capabilities. This type of an analysis thus helps to link the component interactions to the overall physiological functions and how such functions can be affected by genetic parameters and how they respond to environmental conditions.

Recently, with the continued development of technologies and experimental procedures to calculate cellular fluxes using isotopomer data and to carry out quantitative metabolomic measurements on a larger scale [15]–[19], a more complete biochemical characterization of cells has become possible. The approach and analysis presented herein, using the Jacobian to characterize network and cellular level dynamics will benefit from and serve to benefit the utility of these large datasets. Measurements of the fluxome and metabolome at various time points, under different perturbations can be analyzed in terms of overall dynamics and used to validate the model when computed and measured results agree and alternatively used to highlight areas where further revisions are needed when they disagree. This will conceivably add a new dimension to the analysis of metabolism on the genome-scale.

## Materials and Methods

The method developed above was developed, tested, and implemented in Mathematica (Wolfram Research, Chicago, IL) version 5.2. The models analyzed herein: the model of human red cell metabolism [20]–[22], human folate metabolism [23], and yeast glycolysis [24] were implemented in Mathematica.

For each model, a stable steady state was identified by integrating the equations over time until the concentration variables no longer changed (error <1×10^−10^, see Table S1). The Jacobian was then calculated symbolically at that steady state condition.

Temporal decomposition was carried out as described in the Results/Discussion section. Briefly for a general case, a similarity transformation [8] of a square matrix, A, is given by A = DΛD^−1^ in which D is invertible (by definition) and Λ is a diagonal matrix. D is an orthogonal matrix composed of eigenvectors corresponding to the entries of Λ (the eigenvalues). When the Jacobian matrix for a first order differential equation with respect to time is decomposed in this manner, the negative reciprocals of the eigenvalues correspond to the characteristic time scales for the corresponding modes [8] (this is immediately clear upon integration of Equation 4). All three of the models considered here exhibited at least one pair of complex conjugate eigenvalues at the steady states considered, hence the corresponding complex conjugate modes were combined in order to eliminate oscillating motions.

The calculations for the correlations across progressive time scales were carried out as described in Results/Discussion. Once the modal matrix, M^−1^, was calculated, all pairwise angles between the metabolites (columns of the modal matrix) were calculated (see Equation 5). The modal matrix is rank ordered from the fastest (*k* = 1) to the slowest (*k* = *n*) modes. The angles between the columns of the modal matrix were recalculated *n*−1 more times, in which an additional row of the modal matrix is zeroed out at each iteration. For example at the third iteration (*k* = 2), the first two rows of the modal matrix have been zeroed out. The spectrum of correlation cut-off values for pooling were considered from 10% to 99%. Cut-off values in the range 85% to 95% resulted in pooling of variables most consistent with the known pooling structures of the human red cell [2],[5]. A value of 90% was used as the correlation cutoff for the red cell, folate, and yeast glycolysis models. The angle between two zero vectors was classified as undefined and the angle between any zero vector and another vector with at least one non-zero element was defined as 90°. Fragmentation of the pooling structure, in the strictest sense, was identified by any 0 entry (or <∼10^−13^) in the final row of the metabolite modal matrix.

Values for the Gibbs standard free energies of formation for the metabolites in the human red cell model were used from [25].

## Supporting Information

Figure S1 Illustrative example of time scale decomposition. An Illustrative example of modal decomposition for a toy network. The dynamics of 3 reactions involving 6 metabolites is analyzed in terms of the Jacobian matrix. Time scale decomposition is carried out along with simulations and determination of the pooling structure for this toy example.
(0.09 MB PDF)

Figure S2 Standard free energy of formation ratios for metabolites in the human red blood cell. The lower left triangle of the tiled array depicts a matrix with the ratios of the Gibbs free energy of formation between the metabolites in the red blood cell metabolic network. Ratios below 0.85 or above 1.15 were filtered out and not shown. The remaining entries (blackened entries) indicate expected pools if thermodynamic considerations alone determined the behavior of the network in a closed system.
(0.06 MB PDF)

Table S1 Steady state concentrations and fluxes for folate, yeast, and red blood cell.
(0.02 MB XLS)
