# Supplementary material for: Top-Down Analysis of Temporal Hierarchy in Biochemical Reaction Networks
Source: PLoS Comput Biol. 2008 Sep 12;4(9):e1000177. doi: 10.1371/journal.pcbi.1000177 (PMC2518853; doi:10.1371/journal.pcbi.1000177)

Supplementary Figure 2: Standard free energy of formation ratios for metabolites in the human red blood cell. The lower left triangle of the tiled array depicts a matrix with the ratios of the Gibbs free energy of formation between the metabolites in the red blood cell metabolic network. Ratios below 0.85 or above 1.15 were filtered out and not shown. The remaining entries (blackened entries) indicate expected pools if thermodynamic considerations alone determined the behavior of the network in a closed system.

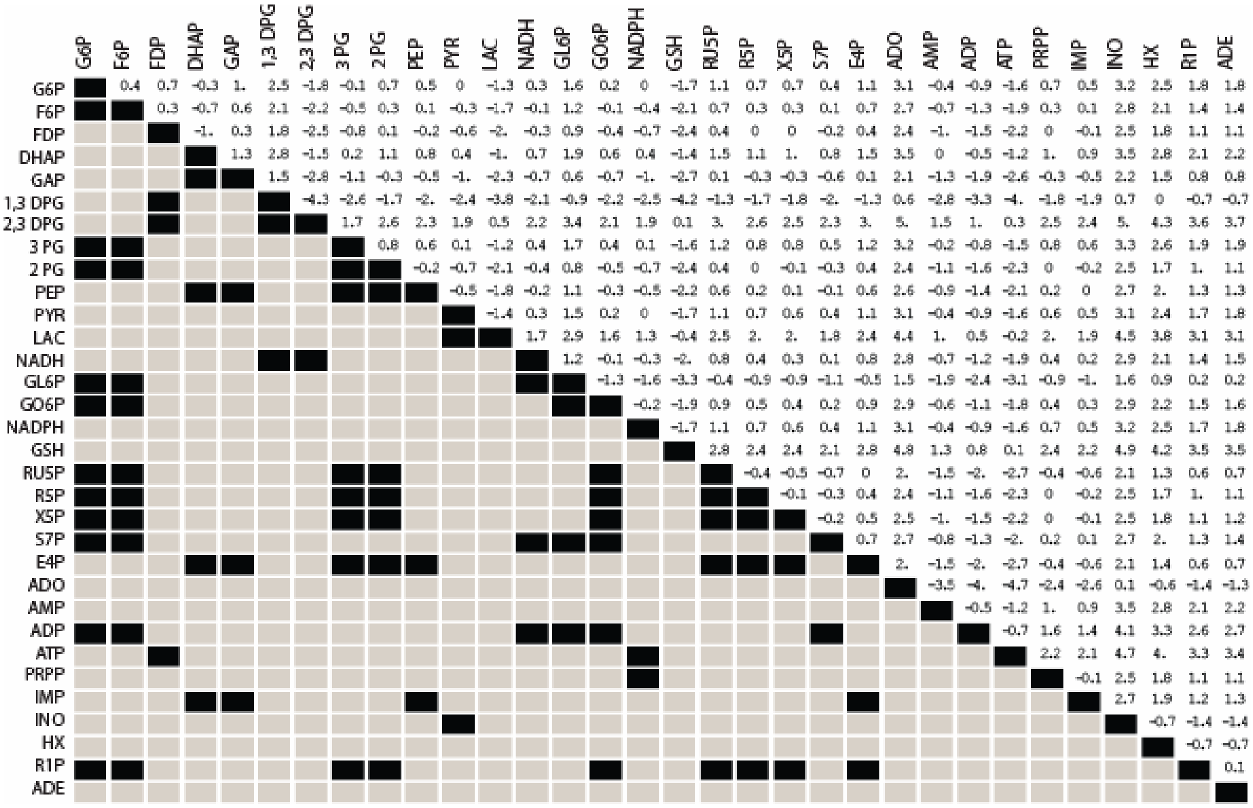

Supplement: Figure S2 — Standard free energy of formation ratios for metabolites in the human red blood cell. The lower left triangle of the tiled array depicts a matrix with the ratios of the Gibbs free energy of formation between the metabolites in the red blood cell metabolic network. Ratios below 0.85 or above 1.15 were filtered out and not shown. The remaining entries (blackened entries) indicate expected pools if thermodynamic considerations alone determined the behavior of the network in a closed system. (0.06 MB PDF) [file pcbi.1000177.s002.pdf]
