# Supplementary material for: Top-Down Analysis of Temporal Hierarchy in Biochemical Reaction Networks
Source: PLoS Comput Biol. 2008 Sep 12;4(9):e1000177. doi: 10.1371/journal.pcbi.1000177 (PMC2518853; doi:10.1371/journal.pcbi.1000177)

Supplement: An Illustrative example of modal decomposition for a toy network.

Consider a simple 'toy' group of reactions given by,

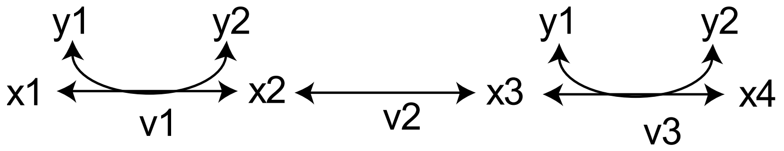

These three reactions and six metabolites can be described by a 6x3 stoichiometric matrix,

	v1	v2	v3
x1	-1	0	0
x2	1	-1	0
x3	0	1	-1
x4	0	0	1
y1	-1	0	-1
y2	1	0	1

Ascribing equilibrium constants to the reactions and forward rate constants, the mass action rate expressions immediately follow.

	Equilibrium constants	Forward rate constants	Rate equations
v1	100	1.3	0.26 x1 - 0.026 x2
v2	2	100000	100000 x2 - 50000 x3
v3	100	0.3	0.06 x3 - 0.006 x4

Having defined the stoichiometric matrix and the rate equations, a particular steady state can be solved for. In this example we set the concentration of x3 to 0.5 mM, thereby fixing the network at a particular steady state given by,

	mM
x1	0.025
x2	0.25
x3	0.5
x4	5.
y1	0.2
y2	2

Defining the deviation variables as $x' = x - x_{ss}$, with x_{ss} referring to a steady state vector. After linearizing around a steady state,

$$\frac{dx'}{dt} = Jx'$$

in which J is the Jacobian. For the above toy system this equation,

$$dx'/dt \quad = \quad \begin{pmatrix} -0.26 & 0.026 & 0 & 0 & -0.0325 & 0.00325 \\ 0.26 & -100000. & 50000 & 0 & 0.0325 & -0.00325 \\ 0 & 100000 & -50000.1 & 0.006 & -0.15 & 0.015 \\ 0 & 0 & 0.06 & -0.006 & 0.15 & -0.015 \\ -0.26 & 0.026 & -0.06 & 0.006 & -0.1825 & 0.01825 \\ 0.26 & -0.026 & 0.06 & -0.006 & 0.1825 & -0.01825 \end{pmatrix} \begin{pmatrix} x1 \\ x2 \\ x3 \\ x4 \\ y1 \\ y2 \end{pmatrix}$$

Applying a similarity transformation to J and calculating the modal matrix (see equations 2 and 3 in the main text) results in,

Time Scales (min)	x1	x2	x3	x4	y1	y2
6.66667×10^{-6}	1.73334×10^{-6}	-1.	0.5	$-2.\times10^{-8}$	7.16668×10^{-7}	-7.16668×10^{-8}
3.00914	1.	0.00101096	0.00101062	-0.00515161	0.25379	-0.025379
5.46163	1.	-0.182652	-0.182652	0.0223978	-0.434944	0.0434944

in which the time scales are the negative reciprocals of the eigenvalues. Note that since the rank of the Jacobian is only 3, there are only 3 modes in this system. From the time scales, one can see that these reactions span about 7 orders of magnitude.

In order to explicitly see visualize the modes, we can plot the relaxation of this systems in response to a perturbation (through integration of equation 4 in the main text).

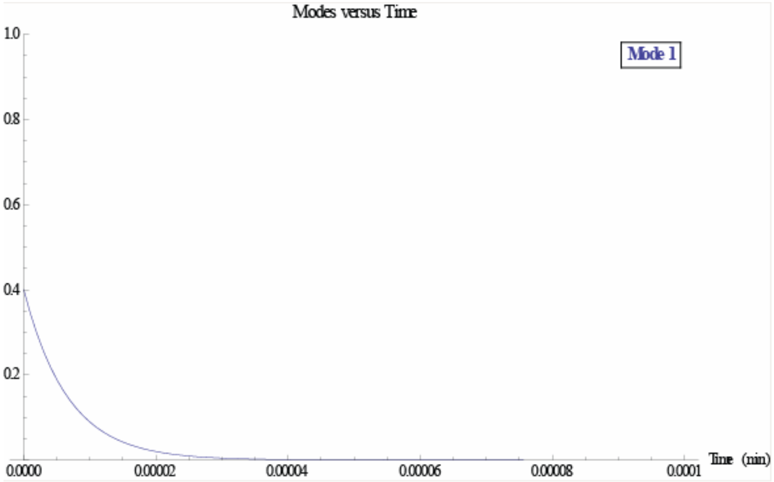

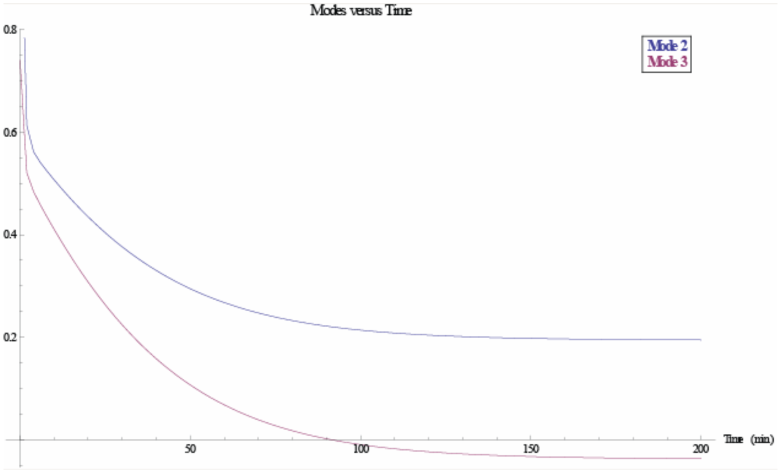

Mode 1 is plotted separately from Modes 2 and 3 because their dynamic responses take place on significantly different time scales. Full relaxation of Mode 1 takes place on the order of milliseconds, whereas full relaxation of Modes 2 and 3 take place on the order of hours.

Due to the small number of variables and simplicity of this example, the pooling structure can be immediately identified in this example. x2 and x3 immediately equilibrate and move together

across all of the time scales. y1 and y2 pool together after the first time scale, finally to be joined by x1 and x4. The corresponding pooling plot can be seen,

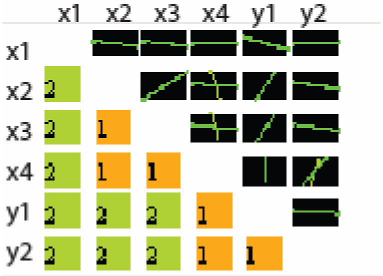

Supplement: Figure S1 — Illustrative example of time scale decomposition. An Illustrative example of modal decomposition for a toy network. The dynamics of 3 reactions involving 6 metabolites is analyzed in terms of the Jacobian matrix. Time scale decomposition is carried out along with simulations and determination of the pooling structure for this toy example. (0.09 MB PDF) [file pcbi.1000177.s001.pdf]
